# Prevalence of Drug–Drug Interactions in Primary Care Prescriptions in Egypt: A Cross-Sectional Retrospective Study

**DOI:** 10.3390/pharmacy11030106

**Published:** 2023-06-18

**Authors:** Khaled Abdelkawy, Maged Kharouba, Khloud Shendy, Omar Abdelmagged, Naira Galal, Mai Tarek, Mohamed Abdelgaied, Amr Y. Zakaria, Sherif Hanafy Mahmoud

**Affiliations:** 1 Faculty of Pharmacy, Kafrelsheikh University, Kafrelsheikh 33516, Egyptomar.abdelfattah@pharm.kfs.edu.eg (O.A.); naiera.galal@pharm.kfs.edu.eg (N.G.); mai.tareq@pharm.kfs.edu.eg (M.T.); 2 Faculty of Pharmacy and Pharmaceutical Sciences, University of Alberta, Edmonton, AB T6G 2E1, Canada; 3 Faculty of Pharmacy and Biotechnology, German University in Cairo, Cairo 11511, Egypt; 4 Faculty of Pharmacy, Horus University, Damietta 34511, Egypt

**Keywords:** drug interactions, Egypt, primary care, community pharmacy, pharmacists

## Abstract

In clinical practice, drug–drug interactions (DDIs) pose significant risks to a large number of patients. Consequently, healthcare providers are required to diligently identify, monitor, and effectively handle these interactions in order to enhance patient outcomes. In Egypt, DDIs are poorly addressed, with no reports for DDIs in primary care. In our cross-sectional, retrospective, observational study, we collected a total of five thousand, eight hundred and twenty prescriptions across eight major governorates in Egypt. Prescriptions were collected over a span of 15 months between 1 June 2021 and 30 September 2022. These prescriptions were analyzed for potential DDIs using the Lexicomp^®^ drug interactions tool. The prevalence of DDIs was found to be 18%, with 22% of the prescriptions having two or more potential DDIs. Moreover, we found 1447 DDIs of categories C (monitoring therapy recommended), D (therapy modification suggested), and X (avoid combination). The most commonly interacting drugs in our study were diclofenac, aspirin, and clopidogrel, while non-steroidal anti-inflammatory drugs (NSAIDs) were the most reported therapeutic class implicated in pharmacologic DDIs. Pharmacodynamic agonistic activity was the most common mechanism of interaction. Therefore, it is crucial to conduct screenings, detect early signs, and closely monitor drug–drug interactions (DDIs) to enhance patients’ overall health outcomes, medication responses, and safety. In this regard, the clinical pharmacist assumes a vital role in implementing these preventive measures.

## 1. Introduction

Adverse drug events (ADEs) refer to unintended effects following drug administration to patients. They are prevailing complications following medication therapy, affecting millions of patients annually and representing 5% of hospital admissions [1]. The outcomes of ADEs encompass increased mortality and morbidity rates, prolonged hospitalization, increased healthcare expenses, and diminished patient adherence with treatment, resulting in suboptimal therapy outcomes and treatment failure.

Drug–drug interactions (DDIs), a subtype of ADEs, arise when two or more medications are administered simultaneously, leading to a potentially altered therapeutic effect. Patient-specific characteristics such as age, sex, genetics, or underlying medical conditions can contribute to DDIs. Moreover, DDIs can occur due to therapy-related factors including dosage, administration formulation, route of administration, and treatment duration. DDI implications may range from minor, non-clinically significant effects to life-threatening conditions. Worldwide, it is not uncommon that patients’ conditions are managed using more than one drug at a time. The complexity of medication therapies and concomitant usage of multiple medications of various pharmacological classes places those patients at higher risk of DDIs. Therefore, the detection of drug interactions is crucial to prevent potentially harmful reactions.

Much like many other countries, Egypt still faces enduring obstacles related to the identification, prevention, and management of DDIs. The scarcity of documentation of patient medication records in most community pharmacies and the absence of DDI checking software or tools has resulted in DDI identification being merely dependent on pharmacists and healthcare professionals’ efforts during patient assessment. Furthermore, busy pharmacy environments with high volumes of prescriptions might result in clinically important DDIs being missed. Recently, significant strides have been made in addressing the issue of DDIs and mitigating their associated risks. These efforts encompass various key aspects, such as enhancing healthcare teams’ and patients’ awareness of DDIs, emphasizing the urgent need to adopt advanced tools and technologies for identifying and preventing these interactions, and introducing a dedicated course focusing mainly on drug interactions in pharmacy schools [2]. A few observational studies focusing on drug utilization and potential DDIs in Egypt have been reported [3,4,5]; however, none of them have studied DDIs in primary care. Therefore, the aim of the current study was to determine the prevalence, nature, and severity of DDIs in primary care through prescriptions presented to community pharmacies across eight governorates in Egypt, with a focus on examining the specific mechanisms behind these interactions.

## 2. Materials and Methods

This was a cross-sectional, retrospective review of patient prescriptions presented to community pharmacies across eight governorates in Egypt, including Kafrelsheikh, Gharbia, Alsharquia, Menoufia, AlQalyubia, Dakahlia, Damietta, and Alexandria. The study was approved by the Ethical Review Committee of Kafrelsheikh University according to the Helsinki declaration (Project identification code: KFS-ERC045; Date of approval: 24 April 2020). All of the collected data were anonymous and did not contain any patient information. Patient informed consent was not required for this study. The study was coordinated by the Clinical Pharmacy Research Center, Faculty of Pharmacy Kafrelsheikh University. Prescriptions were collected over 15 months in the period from 1 June 2021 to 30 September 2022. The collection of prescriptions from community pharmacies was carried out in cooperation with the Faculty of Pharmacy, Kafrelsheikh University. These pharmacies serve as partners in the training and residency program for Pharm D pharmacy students. Specifically, these pharmacies cater to the eight surrounding governorates of Kafrelsheikh, where the majority of pharmacy students are expected to participate in the residency or training program.

All medications listed in each prescription were checked for the potential for DDI using the Lexicomp^®^ software DDI checker module (Lexicomp, Inc., Macedonia, OH, USA). In Lexicomp^®^, each interaction was given an interaction category (A, B, C, D, or X), reflecting both the level of severity and evidence. As stated by the module, category A indicates no known interaction, category B indicates no action needed, category C recommends monitoring therapy while both agents are used concomitantly, category D suggests therapy modification, and category X suggests avoiding the combination of the interacting drugs. In this study, categories C, D, and X were included as they suggest the potential for clinically significant drug interactions. These categories call for therapeutic interventions such as patient monitoring, therapy modification, or avoiding drug combinations. For each reported interaction, the prescription ID, patients’ age and sex, drug name, pharmacological class, clinical indication (if documented), drug class, mechanism of interactions, and recommendations were documented in a drug interaction report form.

Statistical analysis was carried out using Stata software version 15.1 (College Station, TX, USA). Data were summarized using descriptive statistics. Categorical variables were presented as *n* (%). Categorical variables were compared using the Chi-square or Fisher exact test, as appropriate. The level of significance was set at *p*-value < 0.05.

## 3. Results

### 3.1. Number of Interactions per Prescription

**Figure 1:**
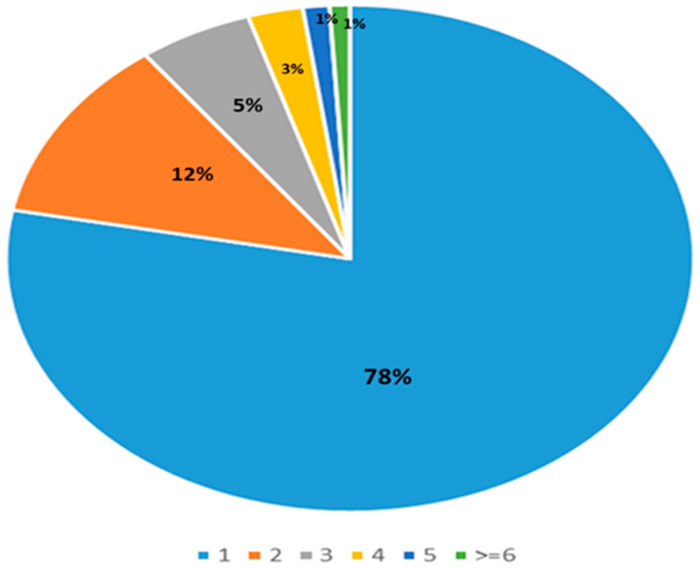
Number of drug–drug interactions per prescription.

A total of 5820 prescriptions were included and assessed for DDIs by Lexicomp^®^. A total of 1447 interactions of categories C, D, or X were found across 1045 prescriptions (18%). The order of reported Lexicomp^®^ categories by the number of interactions was category C, category D, followed then by category X, each representing 48.2%, 28.3%, and 23.4% of all interactions, respectively. Some prescriptions had more than one interaction, as depicted in Figure 1.

### 3.2. Number of Interactions by Patients’ Age and Sex

The reported age range of the prescriptions was 12–85 years. Older adults (aged ≥65 years) represented about 22% of the participants, whereas younger adults (aged 12–18 years) represented only about 5%. In addition, female patients represented 61% of prescriptions, while male patients only represented the remaining 39%. There were no significant differences between males and females in terms of interaction class or mechanism. As shown in Table 1, category X interactions were highly represented in younger adults and adults when compared to older adults, while category C interactions were highly represented in older adults (*p*-value < 0.0001).

### 3.3. Commonly Interacting Drugs

**Figure 2:**
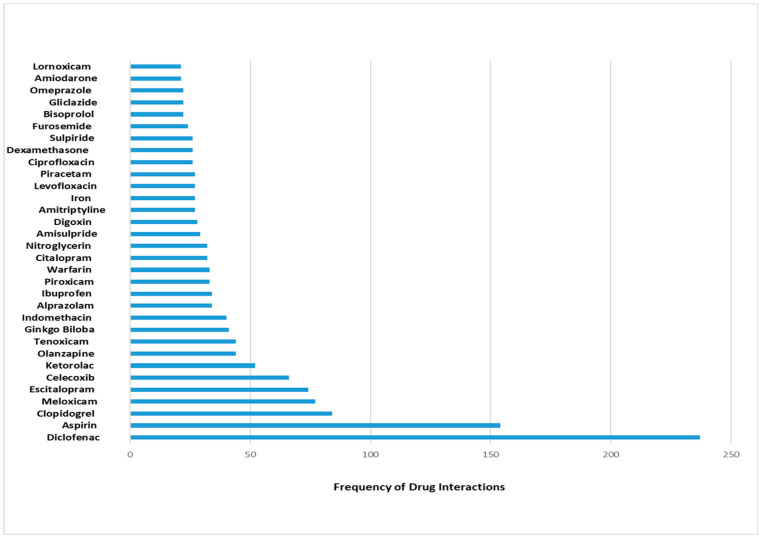
Drugs commonly implicated in interactions in the current study. Drugs with ≥20 interactions were included in this figure.

Figure 2 depicts drugs that were most implicated in DDIs. As shown in this figure, seven drugs were implicated in more than fifty drug interactions. These drugs were diclofenac, aspirin, clopidogrel, meloxicam, escitalopram, celecoxib, and ketorolac, with 237, 154, 84, 77, 74, 66, and 52 interactions, respectively. Both diclofenac and aspirin had interaction frequencies of more than 100 interactions, with diclofenac alone represented about 8% of the reported interactions in this study. The most commonly reported interactions with systemic diclofenac were therapeutic duplication with other NSAIDs such as celecoxib, meloxicam, or naproxen (categories D or X).

### 3.4. Commonly Interacting Pharmacological Classes

**Figure 3:**
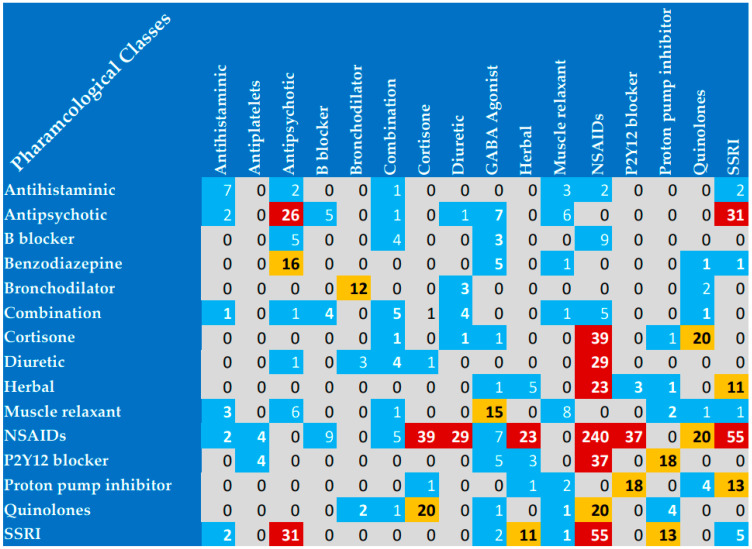
Pharmacological classes commonly implicated in drug interactions in the current study. SSRIs, selective serotonin reuptake inhibitors; NSAIDs, non-steroidal anti-inflammatory drugs; GABA, gamma-aminobutyric acid. Red color, >20 interactions; yellow color, 10–20 interactions; blue color, <10 interactions; grey color, no reported interactions. “Combinations” refer to fixed-dose drug combinations where two or more active ingredients are included in one tablet. For example, Ramipril and Hydrochlorothiazide or Bisoprolol fumarate and Hydrochlorothiazide. Herbals included Ginkgo biloba, green tea, and St. John’s wort.

As shown in Figure 3, NSAIDs were the pharmacological class most commonly implicated in DDIs, with a total of 379 interactions. Most NSAID interactions were reported with other NSAIDs (therapeutic duplication), corticosteroids, selective serotonin reuptake inhibitors (SSRIs), and P2Y12 blockers (antiplatelets e.g., Clopidogrel and Ticagrelor). Additionally, quinolones, antipsychotics, corticosteroids, and SSRIs were highly represented in drug interactions with other pharmacological classes. One interesting finding was that drug interactions attributed to therapeutic duplication were reported with NSAIDs, antipsychotics, antihistaminic, SSRIs, bronchodilators, and muscle relaxants, representing 338 of the reported interactions. Another finding was that the herbal product Ginkgo biloba, commonly prescribed as a circulatory stimulant in Egypt, was implicated in a high number of interactions with SSRIs and NSAIDs.

### 3.5. Lexicomp Categories of Commonly Interacting Pharmacological Classes

**Figure 4:**
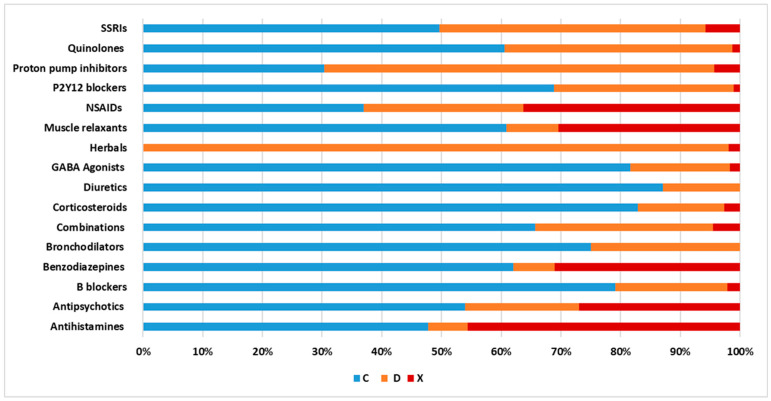
Distribution of the Lexicomp interaction categories by pharmacological class. SSRIs, selective serotonin reuptake inhibitors; NSAIDs, non-steroidal anti-inflammatory drugs; GABA, gamma-aminobutyric acid. Red color, category X; orange color, category D; blue color, category C.

Antihistamines, NSAIDs, benzodiazepines, muscle relaxants, and antipsychotics were the pharmacological classes with most category X interactions (Figure 4), representing 45%, 37%, 32%, 31%, and 27% category X interactions, respectively. On the other hand, category D interactions were highly represented among herbal products and proton pump inhibitors (PPIs), which were implicated in 96% and 65% of the interactions, respectively. Similarly, interactions including “the cardioprotective dose” of aspirin were mostly of categories C or D and were predominantly pharmacodynamic interactions. Most aspirin interactions were found with NSAIDs, sulphonylureas, diuretics, clopidogrel, and piracetam. Meanwhile, regarding herbal products, the main mechanism was pharmacodynamic agonistic activity. The remaining pharmacological classes showed a higher percentage of category C interactions.

### 3.6. Mechanisms of Drug Interactions and Lexicomp Category

Table 2 depicts the mechanisms of the DDIs in each Lexicomp category. The order of the reported mechanisms of interaction by the number of interactions was pharmacodynamic, pharmacokinetic, and mixed (both pharmacodynamics and pharmacokinetics) representing 76.2, 19, and 4.8% of the interactions, respectively. Most interactions that require avoiding combination (category X) were attributed to pharmacodynamic interactions (~94%), mainly causing agonistic activity regarding both efficacy and adverse effects. Aspirin taken at cardioprotective doses produced interactions that were mostly pharmacodynamic. Most aspirin interactions were found with NSAIDs, sulphonylureas, diuretics, clopidogrel, and piracetam. On the other hand, the main pharmacokinetic interaction mechanism that necessitated avoiding combination (category X) was metabolic interaction, as seen with PPIs through the inhibition of cytochrome P450 (CYP) enzymes. Pharmacokinetic interactions were the main mechanism found in fluoroquinolone and proton pump inhibitor (PPI) interactions Absorption pharmacokinetic interactions occurred involving both quinolones and PPIs, whereas metabolic pharmacokinetic interactions were reported involving antipsychotics, corticosteroids, GABA agonists (e.g., benzodiazepines, phenobarbital), PPIs, quinolones, and P2Y12 blockers. Moreover, excretion drug interactions were only reported involving diuretics and NSAIDs.

## 4. Discussion

Understanding the frequency and characteristics of potential drug–drug interactions (DDIs) in Egypt is vital in order to initiate an appropriate preventive healthcare program aimed at averting these interactions. According to our knowledge, this work was the first observational study to investigate the prevalence and mechanisms of potential DDIs in outpatient clinics in Egypt. Our study involved a retrospective collection of prescriptions representing different clinical disorders from different governorates across Egypt, including a major city like Alexandria. In addition, we covered more than a year of prescription collection to ensure seasonal illnesses were captured, such as chest infections in winter and dermatological disorders in summer.

The main findings of the current study demonstrated that the prevalence of potential DDIs represented an estimate of 18% of the collected prescriptions, with about 22% of the prescriptions having two or more interactions, confirming the role of polypharmacy as a potential risk for drug interactions. The utilization of multiple medications (prescription and over-the-counter medications) to treat one or more health conditions, known as polypharmacy, raises the likelihood of experiencing an elevated potential for DDIs. In our cohort, older adults (aged ≥ 65 years) represented about 22% of the participants, whereas younger adults (aged 12–18 years) represented only about 5%. Polypharmacy is common in older adults due to the presence of comorbid conditions that require treatment with multiple medications. As a result, older adults are considered to be at increased risk of DDI and poor outcomes [6,7]. To illustrate, in Australia, a study has reported that 94% of individuals with chronic illnesses aged 65 years or older are prescribed at least five medications per day, increasing the risk of drug interactions [8]. In terms of sex, there were no significant differences between males and females in terms interaction class or mechanism. However, female patients represented 61% of the prescriptions, while male patients only represented the remaining 39%. This highlights the fact that females may have higher propensity for DDIs compared to males. It has been reported that females have an up to 75% higher likelihood of encountering ADRs compared to males. These differences were attributed to polypharmacy, increased drug bioavailability, and heightened sensitivity to medications [9].

In the present study, the most reported interacting drugs were diclofenac, aspirin, and clopidogrel. Specifically, diclofenac was represented in 8% of the reported interactions. Furthermore, the most commonly interacting pharmacological classes were NSAIDs, fluoroquinolones, antipsychotics, corticosteroids, and SSRIs. These findings suggest that healthcare providers should be vigilant when these classes of medications are prescribed. Previous research has exhibited variations in terms of the leading medications that interacted with each other. For example, the most prominent interacting medications in critically ill Egyptian patients were as follows: clopidogrel, aspirin at a low dosage, and atorvastatin [5]. Furthermore, in a study by Bertoli et al., the top drugs involved in the reported DDIs were aspirin, captopril, and corticosteroids [10]. In a study by Oman, the most prescribed and interacting drugs were omeprazole and clindamycin, with incidence rates of more than 25% of interactions, and about 54% of patients experienced at least one potential DDI [11]. In Pakistan, ibuprofen and diclofenac co-administration with levofloxacin was the most common reported interaction, and NSAIDs was the most interacting pharmacological class [12]. These reported differences may be attributed to the differences in the included study populations, regional prescribing patterns, and differences in medication availabilities among countries.

The most encountered drug interaction mechanism was mainly pharmacodynamic agonistic activity, while the most reported pharmacokinetic mechanism was metabolic activity. Similarly, the most documented category of interactions was category C. However, categories D and X were still remarkable (both D and X interactions represented 50% of interactions). Category X drug interactions were highly presented involving NSAIDs, benzodiazepines, muscle relaxants, and antipsychotics, while category D interactions were highly encountered involving herbal products. The most prevalent clinical conditions associated with potential drug–drug interactions (DDIs) included osteoarthritis, diabetes, coagulation disorders, infectious disorders, and depression. Most importantly, osteoarthritis was represented in 463 of the reported interactions, and most of the interactions were due to the duplication of NSAIDs for osteoarthritis management. Therapeutic duplication was an identified issue in the present study. The prescriptions involving two NSAIDs in the same prescription highlight a common medication error in clinical practice in Egypt. Therefore, healthcare providers attending to patients with conditions such as osteoarthritis should exercise heightened vigilance regarding potential drug interactions prior to prescribing medications.

Identifying the prevalence and nature of DDIs in prescriptions will provide an important first step towards optimizing the efforts to improve awareness among healthcare professionals and patients regarding DDIs’ associated risks. The prevalence of DDIs reported in hospitalized patients was lower than what we have seen in our study. To illustrate, in a previous study with similar prescription numbers but conducted on hospitalized patients, DDI incidence was 8.3% in older adults. [6]. In contrast, the prevalence was higher in critically ill patients treated in the ICU. In fact, one potential DDI was documented in more than 50% of patients [13]. Moreover, in Egypt, the prevalence of DDIs in ICU patients was about 53% [5]. This high prevalence underlines the urgent need for the implementation of preventive measures to avoid potential DDIs and stresses the importance of the clinical pharmacist’s role in avoiding potential DDIs in critically ill patients [4]. With regards to primary care, a higher prevalence of potential DDIs was obtained in Brazil [14], but a lower prevalence was documented in Canada [15] compared to our study. In Pakistan, DDIs affected 22.3% of outpatients, which was similar to our finding [12,16]. In the USA, ADEs precipitated by DDIs accounted for 2.8% of hospital admissions every year [8]. Furthermore, in Thailand, a study revealed that the median DDI prevalence rate for hospital admissions was 22.2% [17]. In Egypt, the scarcity of documentation in pharmacy records may have contributed to the increased prevalence of medication errors.

The early detection of DDIs is essential to establish safe medication use. Multiple efforts could be implemented to proactively reduce DDIs, including the incorporation of the patient care process in prescription reviews [18], patient counseling, and the use of electronic health records (EHRs) rather than paper records. Additional training for clinical pharmacists regarding the patient care process will improve the detection of DDIs and improve the quality of the provided care. Following the patient care process is an important step in identifying and addressing actual and potential DDIs [19]. Pharmacists play an important role in preventing DDIs. They are healthcare professionals who are experts in medication therapy and are responsible for ensuring the safe and effective use of medications. Furthermore, technological approaches have provided new opportunities to avoid DDIs by introducing clinical decision support systems (CDSS) [20] that use algorithms and established databases to guide healthcare providers regarding DDIs. EHRs and electronic pharmacy systems generally have a built-in CDSS that prompts healthcare providers with alerts regarding DDIs based on a patient’s drug list. This CDSS checks for interactions using software programs like Medscape^®^, Micromedex^®^, Epocrates^®^, DrugBank^®^, and Lexicomp^®^. This is in addition to the availability of various textbooks that help healthcare professionals recognize and manage DDIs, such as Stockley’s Drug Interactions and Drug Facts and Comparisons [2,21]. The incorporation of CDSSs into physician order entry helps ensure proper medication dosing, the identification of drug duplications, and the detection of DDIs [20]. Finally, adequate patient education is another aspect to consider in overcoming the harmful effects of DDIs. It is highly recommended that healthcare providers enhance patients’ understanding of appropriate drug administration, monitoring of drug therapy, and anticipation of potential drug interactions in terms of their severity, outcomes, and management.

This study’s primary strength lay in its ability to provide valuable data regarding the prevalence of potential drug–drug interactions (DDIs) in Egyptian prescriptions. This valuable information will undoubtedly contribute to enhancing patient safety by offering insights into the characteristics of significant interactions that necessitate prompt intervention to maintain optimal patient care.

This study had several limitations that should be acknowledged. One limitation of this study was that it solely relied on retrospective prescription data collection. As our collected data were solely from the prescriptions provided by the participating pharmacies, our data were limited to what was included in the prescriptions. In terms of patient characteristics, we collected data regarding the age and sex of the patients. However, important participant information such as social history, previous medications, smoking history, lifestyle factors, weight, laboratory values, social history, and other comorbidities were not captured. In addition, we did not record demographic data for prescriptions that did not show any drug interaction. The lack of such data impeded our ability to conduct multivariate analysis to identify predictors associated with DDIs. Furthermore, the study did not track participants for any reported ADEs, which could have provided valuable insights. Another limitation of our study was the absence of data regarding the exact number of pharmacies in each governorate. Despite our best efforts to maintain an equal representation of pharmacies from different governorates, the lack of this specific information poses a potential constraint on the generalizability of our findings. Finally, we did not record the number of drugs per prescription, impeding the accurate determination of the prevalence of polypharmacy among the reviewed prescriptions.

## 5. Conclusions

DDIs are prevalent among primary care prescriptions in Egypt. The drugs most frequently reported to interact were diclofenac, aspirin, and clopidogrel. In addition, herbal products should be used and monitored with caution, as they have the potential to contribute to DDIs. Moreover, therapeutic duplication is a prevalent and recognized significant issue. Therefore, preventive measures are required to overcome the occurrence of DDIs. This includes educating healthcare teams about common DDIs, using DDI checking software, the integration of CDSSs in electronic medical records, and adequate patient counseling. Pharmacists’ participation in these preventive measures is essential. Future studies are needed to implement and evaluate DDI prevention measures to enhance patient safety.

## Figures and Tables

**Table 1:** Mechanisms of drug interactions and Lexicomp^®^ category in relation to age and sex.

		Age			**Sex**	
		Younger Adults	Adults	Older Adults	Male	**Female**
Interaction Class	C	30	479	189	278	420
		48.3%	45%	58.9%	48.9%	47.7%
	D	15	314	81	160	250
		24.2%	29.5%	25.1%	28.2%	28.5%
	X	17	271	51	130	209
		27.4%	25.5%	16%	22.9%	23.8%
Mechanism of Drug Interaction	Mixed	4 6.6%	48 4.4%	18 5.5%	29 5.1%	41 4.5%
	PD	45 73.8%	807 73.7%	240 77.7%	428 75.2%	664 75.7%
	PK	13 19.7%	239 21.9%	53 16.8%	101 19.7%	174 19.8%

Younger adults were aged 12–18 years, older Adults were aged ≥65 years, PK: pharmacokinetics, PD: pharmacodynamics, Mixed: mixed pharmacokinetics and pharmacodynamics, X: category X interactions, D: category D interactions, C: category C interactions.

**Table 2:** Mechanisms of drug interactions by Lexicomp^®^ category.

Lexicomp Category	Mechanism of Drug Interactions			Total Number
	PD	PK	Mixed	
C	538	122	38	698
	77.1%	17.5%	5.44%	
D	246	142	22	410
	60%	34.6%	5.36%	
X	318	11	10	339
	93.9%	3.3%	2.95%	
Total	1102 76.2%	275 19%	70 4.8%	1447

## Data Availability

All relevant data are within the manuscript.
